# 5-(4-Chloro­phen­oxy)-6-isopropyl-3-phenyl-3*H*-1,2,3-triazolo[4,5-*d*]pyrimidin-7(6*H*)-one

**DOI:** 10.1107/S160053680903788X

**Published:** 2009-09-30

**Authors:** Xiao-Hua Zeng, Xiao-Ling Liu, Shou-Heng Deng, Ping Chen, Hong-Mei Wang

**Affiliations:** a Institute of Medicinal Chemistry, YunYang Medical College, ShiYan 442000, People’s Republic of China; b Center of Oncology, People’s Hospital affiliated with YunYang Medical College, Shi Yan 442000, People’s Republic of China

## Abstract

In the title compound, C_19_H_16_ClN_5_O_2_, the triazolopyrimidine ring system is essentially planar, with a maximum displacement of 0.021 (4) Å, and forms dihedral angles of 1.09 (9) and 87.74 (9)° with the phenyl and benzene rings, respectively. Short intra­molecular C—H⋯O and C—H⋯N hydrogen-bonding inter­actions occur within the molecule. In the crystal structure, mol­ecules are linked by inter­molecular C—H⋯O hydrogen bonds into chains parallel to the *b* axis. In addition, π–π stacking inter­actions involving the triazole and pyrimidine rings of adjacent mol­ecules are observed, with centroid–centroid distances of 3.600 (3) Å.

## Related literature

For the biological activity of 8-aza­guanine derivatives, see: Roblin *et al.* (1945 ▶); Ding *et al.* (2004 ▶); Mitchell *et al.* (1950 ▶); Levine *et al.* (1963 ▶); Montgomery *et al.* (1962 ▶); Yamamoto *et al.* (1967 ▶); Bariana (1971 ▶); Holland *et al.* (1975 ▶); For related structures, see: Ferguson *et al.* (1998 ▶); Li *et al.* (2004 ▶); Zhao, Xie *et al.* (2005 ▶); Zhao, Hu *et al.* (2005 ▶); Zhao, Wang & Ding (2005 ▶); Chen & Shi (2006 ▶); Maldonado *et al.* (2006 ▶); Xiao & Shi (2007 ▶); Wang *et al.* (2006 ▶, 2008 ▶); Zeng *et al.* (2006 ▶, 2009 ▶).
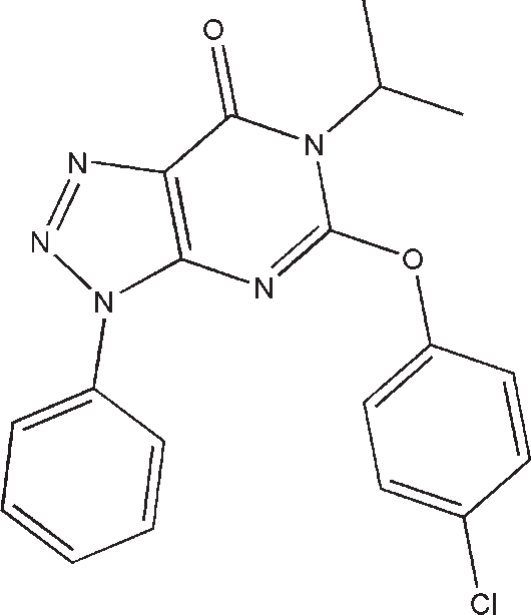


## Experimental

### 

#### Crystal data

C_19_H_16_ClN_5_O_2_
*M* *_r_* = 381.82
Monoclinic, 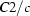

*a* = 16.8429 (3) Å
*b* = 11.7890 (2) Å
*c* = 18.8309 (3) Å
β = 91.737 (2)°
*V* = 3737.36 (11) Å^3^
*Z* = 8
Mo *K*α radiation
μ = 0.23 mm^−1^
*T* = 298 K
0.26 × 0.20 × 0.10 mm

#### Data collection

Bruker SMART CCD area-detector diffractometer
Absorption correction: multi-scan (*SADABS*; Sheldrick, 1996 ▶) *T*_min_ = 0.943, *T*_max_ = 0.978
10890 measured reflections
3290 independent reflections
2697 reflections with *I* > 2σ(*I*)
*R*_int_ = 0.030

#### Refinement

*R*[*F* ^2^ > 2σ(*F* ^2^)] = 0.082
*wR*(*F* ^2^) = 0.188
*S* = 1.20
3290 reflections
246 parameters
H-atom parameters constrained
Δρ_max_ = 0.35 e Å^−3^
Δρ_min_ = −0.27 e Å^−3^

### 

Data collection: *SMART* (Bruker, 2001 ▶); cell refinement: *SAINT* (Bruker, 2001 ▶); data reduction: *SAINT*; program(s) used to solve structure: *SHELXS97* (Sheldrick, 2008 ▶); program(s) used to refine structure: *SHELXL97* (Sheldrick, 2008 ▶); molecular graphics: *PLATON* (Spek, 2009 ▶); software used to prepare material for publication: *SHELXTL* (Sheldrick, 2008 ▶).

## Supplementary Material

Crystal structure: contains datablocks global, I. DOI: 10.1107/S160053680903788X/rz2362sup1.cif

Structure factors: contains datablocks I. DOI: 10.1107/S160053680903788X/rz2362Isup2.hkl

Additional supplementary materials:  crystallographic information; 3D view; checkCIF report

## Figures and Tables

**Table 1:** Hydrogen-bond geometry (Å, °)

*D*—H⋯*A*	*D*—H	H⋯*A*	*D*⋯*A*	*D*—H⋯*A*
C17—H17⋯O2	0.98	2.16	2.679 (4)	112
C6—H6⋯N4	0.93	2.36	3.013 (4)	127
C4—H4⋯O1^i^	0.93	2.46	3.317 (5)	154

Symmetry code: (i) 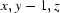.

**Fig. 1.**
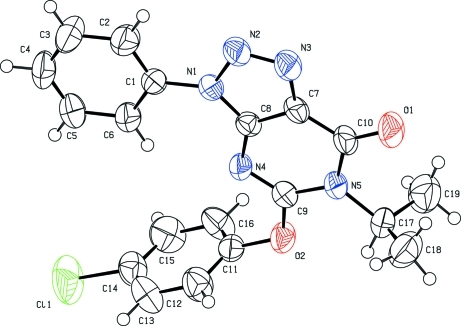
View of the molecule of showing the atom-labeling scheme. Displacement ellipsoids are drawn at 50% probability level. H-atoms are represented by circles of arbitrary size.

## supplementary crystallographic information

### Comment

The derivatives of heterocycles containing the 8-azaguanine system, which are well known bioisosteres of guanine, are of great importance because of their remarkable biological properties. Some of these activities include antimicrobial or antifungal activities (Roblin *et al.*, 1945; Ding *et al.*, 2004), encephaloma cell inhibitor activity (Mitchell *et al.*, 1950; Levine *et al.*, 1963), antileukemie activity (Montgomery *et al.*, 1962), hypersusceptibility inhibitor activity and acesodyne activity (Yamamoto *et al.*, 1967; Bariana, 1971; Holland *et al.*, 1975).

In recent years, we have been engaged in the preparation of the derivatives of 8-azaguanine *via* aza-Wittig reaction of /b-ethoxycarbonyl iminophosphorane with aromatic isocyanate (Zhao, Xie *et al.*, 2005). As a continuation of our research for new biologically active heterocycles, the title compound was obtained from /b-ethoxycarbonyl iminophosphorane with alphalic isocyanate, and structurally characterized in this context.

In the title compound (Fig. 1), bond lengths and angles within the triazolopyrimidinone ring system are in good agreement with those observed for closely related structures (Zhao, Hu *et al.*, 2005; Zhao, Wang & Ding, 2005). As reported for related compounds (Ferguson *et al.*, 1998; Li *et al.*, 2004; Maldonado *et al.*, 2006; Zeng *et al.*, 2006, 2009; Wang *et al.*, 2006, 2008; Xiao *et al.*, 2007; Chen & Shi, 2006), all ring atoms in the 1,2,3-triazolo[4,5-*d*]pyrimidine ring system are essentially coplanar (maximum deviation 0.021 (4) Å for atom C10), indicating that the moiety is a conjugate system. The dihedral angles it forms with the C1–C6 and C11–C16 phenyl and benzene rings are 1.09 (9) and 87.74 (9)°, respectively.

There exist two intramolecular C—H···O and C—H···N hydrogen bonding interactions (Table 1) stabilizing the molecular conformation. The crystal packing is stabilized by intermolecular C—H···O hydrogen bonds forming chains parallel to the *b* axis, and by π–π stacking interactions occurring between adjacent triazole and pyrimidine rings, with centroid-to-centroid distances of 3.600 (3) Å.

### Experimental

To the solution of carbodiimide in CH_2_Cl_2_/CH_3_CN (1:4 *v*/*v*, 15 ml) prepared according to the literature method (Zeng *et al.*, 2006), 4-chlorophenol (3 mmol) and excess K_2_CO_3_ were added, and the reaction mixture was stirred for 12 h. The solvent was removed under reduced pressure and the residue was recrystallized from EtOH to give the title compound (yield 92%; m.p. 459 K). Elemental analysis: calculated for C_19_H_16_ClN_5_O_2_: C, 59.77; H, 4.22; N, 18.34%. Found: C, 58.62; H, 4.48; N, 17.83%. Crystals suitable for single crystal X-ray diffraction analysis were obtained by slow evaporation of a hexane/dichloromethane (1:3 *v*/*v*) solution at room temperature.

### Refinement

H atoms were placed at calculated positions and treated as riding atoms, with C—H = 0.93–0.98 Å, and *U*_iso_(H) = 1.2*U*_eq_(C) or 1.5*U*_eq_(C) for methyl H atoms.

### Crystal data

**Table:**

C_19_H_16_ClN_5_O_2_	*F*(000) = 1584
*M**_r_* = 381.82	*D*_x_ = 1.357 Mg m^−^^3^
Monoclinic, *C*2/*c*	Mo *K*α radiation, λ = 0.71073 Å
Hall symbol: -C 2yc	Cell parameters from 2541 reflections
*a* = 16.8429 (3) Å	θ = 2.4–22.6°
*b* = 11.7890 (2) Å	µ = 0.23 mm^−^^1^
*c* = 18.8309 (3) Å	*T* = 298 K
β = 91.737 (2)°	Block, colourless
*V* = 3737.36 (11) Å^3^	0.26 × 0.20 × 0.10 mm
*Z* = 8	

### Data collection

**Table:**

Bruker SMART CCD area-detector diffractometer	3290 independent reflections
Radiation source: fine-focus sealed tube	2697 reflections with *I* > 2σ(*I*)
graphite	*R*_int_ = 0.030
φ and ω scans	θ_max_ = 25.0°, θ_min_ = 2.1°
Absorption correction: multi-scan (*SADABS*; Sheldrick, 1996)	*h* = −20→19
*T*_min_ = 0.943, *T*_max_ = 0.978	*k* = −11→14
10890 measured reflections	*l* = −22→22

### Refinement

**Table:**

Refinement on *F*^2^	Primary atom site location: structure-invariant direct methods
Least-squares matrix: full	Secondary atom site location: difference Fourier map
*R*[*F*^2^ > 2σ(*F*^2^)] = 0.082	Hydrogen site location: inferred from neighbouring sites
*wR*(*F*^2^) = 0.188	H-atom parameters constrained
*S* = 1.20	*w* = 1/[σ^2^(*F*_o_^2^) + (0.064*P*)^2^ + 5.7684*P*] where *P* = (*F*_o_^2^ + 2*F*_c_^2^)/3
3290 reflections	(Δ/σ)_max_ < 0.001
246 parameters	Δρ_max_ = 0.35 e Å^−^^3^
0 restraints	Δρ_min_ = −0.27 e Å^−^^3^

### Special details

**Table:**

Geometry. All e.s.d.'s (except the e.s.d. in the dihedral angle between two l.s. planes) are estimated using the full covariance matrix. The cell e.s.d.'s are taken into account individually in the estimation of e.s.d.'s in distances, angles and torsion angles; correlations between e.s.d.'s in cell parameters are only used when they are defined by crystal symmetry. An approximate (isotropic) treatment of cell e.s.d.'s is used for estimating e.s.d.'s involving l.s. planes.
Refinement. Refinement of *F*^2^ against ALL reflections. The weighted *R*-factor *wR* and goodness of fit *S* are based on *F*^2^, conventional *R*-factors *R* are based on *F*, with *F* set to zero for negative *F*^2^. The threshold expression of *F*^2^ > σ(*F*^2^) is used only for calculating *R*-factors(gt) *etc*. and is not relevant to the choice of reflections for refinement. *R*-factors based on *F*^2^ are statistically about twice as large as those based on *F*, and *R*- factors based on ALL data will be even larger.

### Fractional atomic coordinates and isotropic or equivalent isotropic displacement parameters (Å^2^)

**Table:**

	*x*	*y*	*z*	*U*_iso_*/*U*_eq_	
C1	−0.09358 (19)	0.1023 (3)	0.18512 (17)	0.0439 (8)	
C2	−0.1566 (2)	0.0468 (3)	0.2139 (2)	0.0642 (11)	
H2	−0.1984	0.0882	0.2319	0.077*	
C3	−0.1583 (3)	−0.0702 (3)	0.2161 (2)	0.0737 (12)	
H3	−0.2009	−0.1075	0.2360	0.088*	
C4	−0.0973 (3)	−0.1310 (3)	0.1891 (2)	0.0689 (12)	
H4	−0.0982	−0.2099	0.1903	0.083*	
C5	−0.0348 (3)	−0.0757 (3)	0.1601 (2)	0.0673 (11)	
H5	0.0067	−0.1175	0.1417	0.081*	
C6	−0.0322 (2)	0.0415 (3)	0.1578 (2)	0.0560 (10)	
H6	0.0105	0.0785	0.1379	0.067*	
C7	−0.06815 (19)	0.4035 (3)	0.17494 (18)	0.0453 (8)	
C8	−0.03822 (18)	0.2986 (3)	0.16141 (16)	0.0397 (7)	
C9	0.0704 (2)	0.3664 (3)	0.11400 (18)	0.0462 (8)	
C10	−0.0258 (2)	0.5037 (3)	0.15489 (19)	0.0510 (9)	
C11	0.16545 (19)	0.2430 (3)	0.06917 (19)	0.0476 (8)	
C12	0.2136 (2)	0.1916 (3)	0.1182 (2)	0.0666 (11)	
H12	0.2299	0.2299	0.1592	0.080*	
C13	0.2380 (3)	0.0818 (4)	0.1064 (2)	0.0727 (12)	
H13	0.2708	0.0449	0.1396	0.087*	
C14	0.2136 (2)	0.0283 (3)	0.0458 (2)	0.0618 (11)	
C15	0.1683 (3)	0.0821 (4)	−0.0042 (2)	0.0762 (13)	
H15	0.1541	0.0454	−0.0465	0.091*	
C16	0.1431 (2)	0.1920 (4)	0.0077 (2)	0.0687 (11)	
H16	0.1114	0.2299	−0.0260	0.082*	
C17	0.1015 (2)	0.5730 (3)	0.1032 (2)	0.0568 (10)	
H17	0.1501	0.5378	0.0864	0.068*	
C18	0.1260 (3)	0.6448 (4)	0.1659 (3)	0.0860 (14)	
H18A	0.0816	0.6893	0.1804	0.129*	
H18B	0.1685	0.6943	0.1530	0.129*	
H18C	0.1436	0.5968	0.2044	0.129*	
C19	0.0657 (3)	0.6376 (4)	0.0409 (3)	0.0867 (14)	
H19A	0.0589	0.5875	0.0010	0.130*	
H19B	0.1005	0.6987	0.0287	0.130*	
H19C	0.0151	0.6678	0.0534	0.130*	
Cl1	0.24101 (10)	−0.11289 (10)	0.03223 (8)	0.1080 (6)	
N1	−0.09221 (15)	0.2236 (2)	0.18491 (14)	0.0435 (7)	
N2	−0.15423 (17)	0.2844 (3)	0.21305 (17)	0.0555 (8)	
N3	−0.13910 (18)	0.3917 (3)	0.20656 (17)	0.0562 (8)	
N4	0.03181 (15)	0.2756 (2)	0.13053 (15)	0.0451 (7)	
N5	0.04809 (16)	0.4770 (2)	0.12434 (15)	0.0470 (7)	
O1	−0.04809 (17)	0.6008 (2)	0.16009 (17)	0.0747 (9)	
O2	0.14044 (14)	0.35551 (19)	0.08206 (14)	0.0595 (7)	

### Atomic displacement parameters (Å^2^)

**Table:**

	*U*^11^	*U*^22^	*U*^33^	*U*^12^	*U*^13^	*U*^23^
C1	0.0430 (18)	0.0388 (19)	0.0495 (19)	0.0002 (15)	−0.0048 (15)	−0.0037 (15)
C2	0.058 (2)	0.051 (2)	0.084 (3)	−0.0023 (19)	0.011 (2)	−0.005 (2)
C3	0.084 (3)	0.048 (2)	0.091 (3)	−0.017 (2)	0.020 (3)	−0.002 (2)
C4	0.095 (3)	0.034 (2)	0.077 (3)	−0.002 (2)	−0.004 (2)	0.0014 (19)
C5	0.073 (3)	0.042 (2)	0.087 (3)	0.013 (2)	0.003 (2)	−0.003 (2)
C6	0.053 (2)	0.041 (2)	0.074 (3)	0.0024 (17)	0.0059 (18)	0.0014 (18)
C7	0.0420 (18)	0.0389 (19)	0.055 (2)	0.0124 (15)	0.0036 (15)	−0.0041 (15)
C8	0.0373 (17)	0.0401 (19)	0.0417 (17)	0.0047 (14)	0.0002 (14)	−0.0037 (14)
C9	0.0433 (19)	0.042 (2)	0.053 (2)	0.0054 (15)	0.0015 (15)	0.0000 (16)
C10	0.055 (2)	0.0335 (19)	0.064 (2)	0.0098 (16)	−0.0051 (17)	−0.0073 (16)
C11	0.0412 (18)	0.041 (2)	0.062 (2)	0.0016 (15)	0.0164 (16)	0.0024 (17)
C12	0.075 (3)	0.056 (2)	0.069 (3)	0.010 (2)	−0.004 (2)	−0.010 (2)
C13	0.081 (3)	0.069 (3)	0.069 (3)	0.029 (2)	0.000 (2)	0.008 (2)
C14	0.066 (2)	0.051 (2)	0.070 (3)	0.0115 (19)	0.026 (2)	0.001 (2)
C15	0.085 (3)	0.074 (3)	0.070 (3)	0.008 (2)	−0.001 (2)	−0.025 (2)
C16	0.066 (3)	0.070 (3)	0.070 (3)	0.024 (2)	−0.006 (2)	−0.006 (2)
C17	0.057 (2)	0.0365 (19)	0.077 (3)	−0.0035 (17)	0.0037 (19)	0.0052 (18)
C18	0.091 (3)	0.068 (3)	0.098 (3)	−0.026 (3)	−0.012 (3)	0.003 (3)
C19	0.098 (4)	0.073 (3)	0.089 (3)	−0.009 (3)	−0.005 (3)	0.020 (3)
Cl1	0.1354 (12)	0.0544 (8)	0.1367 (12)	0.0263 (7)	0.0429 (10)	−0.0117 (7)
N1	0.0396 (15)	0.0378 (15)	0.0533 (16)	0.0061 (12)	0.0041 (12)	−0.0042 (12)
N2	0.0470 (17)	0.0483 (19)	0.072 (2)	0.0101 (14)	0.0133 (15)	0.0009 (15)
N3	0.0543 (18)	0.0444 (19)	0.071 (2)	0.0108 (14)	0.0119 (15)	−0.0055 (15)
N4	0.0434 (16)	0.0315 (15)	0.0606 (17)	0.0021 (12)	0.0067 (13)	0.0009 (13)
N5	0.0459 (16)	0.0328 (15)	0.0624 (18)	0.0066 (12)	0.0040 (13)	−0.0031 (13)
O1	0.0739 (19)	0.0358 (15)	0.116 (2)	0.0105 (13)	0.0193 (17)	−0.0094 (14)
O2	0.0534 (15)	0.0370 (14)	0.0896 (19)	0.0029 (11)	0.0258 (13)	0.0010 (12)

### Geometric parameters (Å, °)

**Table:**

C1—C6	1.371 (5)	C11—C12	1.353 (5)
C1—C2	1.372 (5)	C11—O2	1.414 (4)
C1—N1	1.431 (4)	C12—C13	1.378 (6)
C2—C3	1.380 (5)	C12—H12	0.9300
C2—H2	0.9300	C13—C14	1.357 (6)
C3—C4	1.365 (6)	C13—H13	0.9300
C3—H3	0.9300	C14—C15	1.352 (6)
C4—C5	1.366 (6)	C14—Cl1	1.749 (4)
C4—H4	0.9300	C15—C16	1.384 (6)
C5—C6	1.383 (5)	C15—H15	0.9300
C5—H5	0.9300	C16—H16	0.9300
C6—H6	0.9300	C17—C18	1.500 (6)
C7—N3	1.358 (4)	C17—N5	1.506 (4)
C7—C8	1.362 (4)	C17—C19	1.509 (6)
C7—C10	1.436 (5)	C17—H17	0.9800
C8—N1	1.352 (4)	C18—H18A	0.9600
C8—N4	1.358 (4)	C18—H18B	0.9600
C9—N4	1.296 (4)	C18—H18C	0.9600
C9—O2	1.346 (4)	C19—H19A	0.9600
C9—N5	1.372 (4)	C19—H19B	0.9600
C10—O1	1.210 (4)	C19—H19C	0.9600
C10—N5	1.422 (4)	N1—N2	1.385 (4)
C11—C16	1.348 (5)	N2—N3	1.297 (4)
			
C6—C1—C2	120.0 (3)	C12—C13—H13	120.4
C6—C1—N1	120.6 (3)	C15—C14—C13	121.2 (4)
C2—C1—N1	119.4 (3)	C15—C14—Cl1	119.4 (3)
C1—C2—C3	120.4 (4)	C13—C14—Cl1	119.4 (3)
C1—C2—H2	119.8	C14—C15—C16	119.7 (4)
C3—C2—H2	119.8	C14—C15—H15	120.2
C4—C3—C2	119.8 (4)	C16—C15—H15	120.2
C4—C3—H3	120.1	C11—C16—C15	118.6 (4)
C2—C3—H3	120.1	C11—C16—H16	120.7
C3—C4—C5	119.8 (4)	C15—C16—H16	120.7
C3—C4—H4	120.1	C18—C17—N5	111.7 (3)
C5—C4—H4	120.1	C18—C17—C19	114.9 (4)
C4—C5—C6	120.9 (4)	N5—C17—C19	111.0 (3)
C4—C5—H5	119.5	C18—C17—H17	106.2
C6—C5—H5	119.5	N5—C17—H17	106.2
C1—C6—C5	119.1 (4)	C19—C17—H17	106.2
C1—C6—H6	120.5	C17—C18—H18A	109.5
C5—C6—H6	120.5	C17—C18—H18B	109.5
N3—C7—C8	109.0 (3)	H18A—C18—H18B	109.5
N3—C7—C10	130.5 (3)	C17—C18—H18C	109.5
C8—C7—C10	120.5 (3)	H18A—C18—H18C	109.5
N1—C8—N4	127.7 (3)	H18B—C18—H18C	109.5
N1—C8—C7	106.0 (3)	C17—C19—H19A	109.5
N4—C8—C7	126.3 (3)	C17—C19—H19B	109.5
N4—C9—O2	118.8 (3)	H19A—C19—H19B	109.5
N4—C9—N5	127.5 (3)	C17—C19—H19C	109.5
O2—C9—N5	113.7 (3)	H19A—C19—H19C	109.5
O1—C10—N5	121.3 (3)	H19B—C19—H19C	109.5
O1—C10—C7	126.9 (3)	C8—N1—N2	108.0 (3)
N5—C10—C7	111.8 (3)	C8—N1—C1	131.7 (3)
C16—C11—C12	122.1 (4)	N2—N1—C1	120.2 (3)
C16—C11—O2	119.3 (3)	N3—N2—N1	108.4 (3)
C12—C11—O2	118.6 (3)	N2—N3—C7	108.6 (3)
C11—C12—C13	119.1 (4)	C9—N4—C8	112.7 (3)
C11—C12—H12	120.4	C9—N5—C10	121.0 (3)
C13—C12—H12	120.4	C9—N5—C17	120.5 (3)
C14—C13—C12	119.2 (4)	C10—N5—C17	118.5 (3)
C14—C13—H13	120.4	C9—O2—C11	115.8 (3)
			
C6—C1—C2—C3	0.7 (6)	C6—C1—N1—C8	−0.1 (5)
N1—C1—C2—C3	−178.5 (4)	C2—C1—N1—C8	179.0 (3)
C1—C2—C3—C4	−0.6 (7)	C6—C1—N1—N2	−179.6 (3)
C2—C3—C4—C5	0.2 (7)	C2—C1—N1—N2	−0.4 (5)
C3—C4—C5—C6	0.1 (7)	C8—N1—N2—N3	0.4 (4)
C2—C1—C6—C5	−0.4 (5)	C1—N1—N2—N3	−180.0 (3)
N1—C1—C6—C5	178.7 (3)	N1—N2—N3—C7	−0.2 (4)
C4—C5—C6—C1	0.0 (6)	C8—C7—N3—N2	−0.1 (4)
N3—C7—C8—N1	0.3 (4)	C10—C7—N3—N2	178.8 (4)
C10—C7—C8—N1	−178.6 (3)	O2—C9—N4—C8	−179.1 (3)
N3—C7—C8—N4	−179.7 (3)	N5—C9—N4—C8	0.0 (5)
C10—C7—C8—N4	1.4 (5)	N1—C8—N4—C9	−179.9 (3)
N3—C7—C10—O1	−3.4 (6)	C7—C8—N4—C9	0.1 (5)
C8—C7—C10—O1	175.3 (4)	N4—C9—N5—C10	−1.6 (5)
N3—C7—C10—N5	178.6 (3)	O2—C9—N5—C10	177.5 (3)
C8—C7—C10—N5	−2.7 (5)	N4—C9—N5—C17	178.6 (3)
C16—C11—C12—C13	−2.7 (6)	O2—C9—N5—C17	−2.3 (4)
O2—C11—C12—C13	179.7 (3)	O1—C10—N5—C9	−175.3 (3)
C11—C12—C13—C14	0.4 (6)	C7—C10—N5—C9	2.8 (4)
C12—C13—C14—C15	2.5 (7)	O1—C10—N5—C17	4.4 (5)
C12—C13—C14—Cl1	−177.4 (3)	C7—C10—N5—C17	−177.4 (3)
C13—C14—C15—C16	−3.1 (7)	C18—C17—N5—C9	−119.2 (4)
Cl1—C14—C15—C16	176.8 (3)	C19—C17—N5—C9	111.3 (4)
C12—C11—C16—C15	2.2 (6)	C18—C17—N5—C10	61.1 (4)
O2—C11—C16—C15	179.8 (4)	C19—C17—N5—C10	−68.5 (4)
C14—C15—C16—C11	0.7 (7)	N4—C9—O2—C11	0.2 (5)
N4—C8—N1—N2	179.5 (3)	N5—C9—O2—C11	−179.0 (3)
C7—C8—N1—N2	−0.5 (3)	C16—C11—O2—C9	89.1 (4)
N4—C8—N1—C1	0.0 (6)	C12—C11—O2—C9	−93.2 (4)
C7—C8—N1—C1	−180.0 (3)		

### Hydrogen-bond geometry (Å, °)

**Table:**

*D*—H···*A*	*D*—H	H···*A*	*D*···*A*	*D*—H···*A*
C17—H17···O2	0.98	2.16	2.679 (4)	112
C6—H6···N4	0.93	2.36	3.013 (4)	127
C4—H4···O1^i^	0.93	2.46	3.317 (5)	154

Symmetry codes: (i) *x*, *y*−1, *z*.
